# Basic studies toward ultrafast soft x-ray photoelectron diffraction; its application to probing local structure in iodobenzene molecules

**DOI:** 10.1063/4.0000141

**Published:** 2022-04-27

**Authors:** T. Teramoto, S. Minemoto, T. Majima, T. Mizuno, J. H. Mun, A. Yagishita, P. Decleva, S. Tsuru

**Affiliations:** 1 Institute for Radiation Sciences, Osaka University, 1-1 Machikaneyama-cho, Toyonaka, Osaka 560-0043, Japan; 2 Department of Physics, Graduate School of Science, The University of Tokyo, 7-3-1 Hongo, Bunkyo-ku, Tokyo 113-0033, Japan; 3 Department of Nuclear Engineering, Kyoto University, Kyoto 615-8540, Japan; 4 Institute for Solid State Physics, The University of Tokyo, 5-1-5 Kashiwanoha, Kashiwa, Chiba 277-8581, Japan; 5 Center for Attosecond Science and Technology, Max Planck POSTECH/KOREA Research Initiative, 77 Cheongam-Ro, Nam-gu, Pohang, Gyeongbuk 37673, South Korea; 6 Institute of Materials Structure Science, KEK, 1-1 Oho, Tsukuba, Ibaraki 305-0801, Japan; 7 CNR IOM and DSCF, Università degli Studi di Trieste, Via L. Giorgieri 1, I-34127 Trieste, Italy; 8 Lehrstuhl für Theoretische Chemie, Ruhr-Universität Bochum, D-44780 Bochum, Germany

## Abstract

Ultrafast x-ray photoelectron diffraction (UXPD) for free molecules has a promising potential to probe the local structures of the molecules in an element-specific fashion. Our UXPD scheme consists of three steps: (1) near-infrared laser (NIR) with ns pulse duration aligns sample molecules, (2) ultra-violet laser with fs pulse duration pumps the aligned molecules, and (3) soft x-ray free-electron laser (SXFEL) with fs pulse duration probes the molecules by measuring x-ray photoelectron diffraction (XPD) profiles. Employing steps of (1) and (3), we have measured *I* 3*d* XPD profiles from ground state iodobenzene aligned by the NIR laser with the SXFEL. Then, we have intensively calculated *I* 3*d* XPD profiles with density functional theory, taking degrees of alignments of the molecules into account, to extract a distance between *C* and *I* atoms in iodobenzene from the experimental *I* 3*d* XPD profiles. Although we have failed to determine the distance from the comparison between the experimental and theoretical results, we have succeeded in concluding that the degeneracies of the initial state eliminate the sensitivity on molecular structure in the *I* 3*d* XPD profiles. Thus, the observation of fine structures in the XPD profiles could be expected, if a nondegenerate molecular orbital is selected for a probe of UXPD. Finally, we have summarized our criteria to perform UXPD successfully: (1) to use SXFEL, (2) to prepare sample molecules with the degree of alignment higher than 0.8, and (3) to select a photoemission process from a nondegenerate inner-shell orbital of sample molecules.

## I. INTRODUCTION

Photoelectron angular distributions (PADs) from isolated molecules have been measured for an increasing variety of purposes, including examining details of electron correlation and photoemission dynamics, demonstrating the electron diffraction as a structural probe of single molecules and probing time-resolved photochemical reactions (Refs. 1–5 and references therein). For such PAD studies, we found that molecular frame photoelectron angular distributions (MFPADs) for the core–shell photoelectrons, with an energy of >100 eV, are less influenced by details of molecular potentials; thus, the MFPADs are well described using an x-ray photoelectron diffraction (XPD) picture.^6^ Theoretically, photoelectron diffraction is a consequence of interference between a directly emitted photoelectron wave and elastically scattered waves by surrounding atoms. Thus, the richly structured MFPAD or XPD profiles might provide a means of determining molecular structure in an element-specific fashion. Surface structural analysis based on the XPD theory has been widely developed.^7–11^ Moreover, several methodologies for the structural analysis have been applied, such as the trial-and-error method,^12^ the real-space triangular method,^13^ and the holography^14^ in the surface science field. In the latter two methods, an XPD profile in momentum space is inversely transformed into an atomic position map in real space via Fourier transform with some corrections and modifications. Nevertheless, these ambitious methods are not applicable to the XPD profiles from the isolated molecules. This inapplicability is because the scattering amplitudes are not isotropic, and phase shifts of the amplitudes are strongly dependent on the scattering processes. Hence, we adopted the trial-and-error method to determine structures of molecules in the electronic ground state from our experimental XPD profiles.^15^ Then, for linear CO_2_, bent NO_2_, planar BF_3_, and prolate symmetric top CH_3_F molecules, our method confirmed that bond lengths and angles can be determined with a resolution of less than 0.1 Å and 10°, respectively. Therefore, on the basis of these achievements, we propose an ultrafast XPD (UXPD) scheme using soft x-ray free-electron lasers (SXFELs) to observe experimentally ultrafast rather simple photochemical reactions, e.g., dissociations, eliminations, and isomerization. Meanwhile, with the advent of femtosecond lasers and x-ray free-electron lasers (XFELs), monitoring ultrafast molecular transformations, such as chemical reactions and phase transitions, has been possible for a decade.^16,17^

Electron and x-ray diffraction are the two principal experimental probes for molecular structure determination. Moreover, UXPD has been suggested as a promising probe by a few teams, including ours, for having the element-specific fashion. Therefore, several proposals^15,18–21^ and test experiments^22–26^ for the UXPD have been published. However, results on the transient structure of molecules during photochemical reactions have not yet been reported, except for the recent paper^27^ on the breakup process in O_2_ molecules, applying SXFEL-pump and SXFEL-probe measurements. Meanwhile, we have extracted structure of the I_2_ molecules in the electronic ground state using the SXFEL probe.^26^ The ultrafast dynamics of O_2_ molecules have been obtained through both triple coincidence and high-repetition SXFEL pulse capabilities.^27^ However, triple coincidence rates are extremely low compared with single photoelectron detection rates, and the processing of triple coincidence data is cumbersome compared with direct measurement of a single photoelectron momentum image. Therefore, the outline of our UXPD scheme is as follows: (1) a near-infrared (NIR) laser pulse with ns duration aligns sample molecules, (2) an optical pulse with fs duration excites aligned sample molecules, and (3) SXFEL with fs pulse width probes the molecules by measuring 2D x-ray photoelectron image with velocity map imaging (VMI) spectrometer.^25,26^

In this paper, as a feasibility study toward the UXPD, we report *I* 3*d* XPD profiles from iodobenzene molecules (IPh) aligned with a Nd:YAG laser. These molecules were obtained using SXFEL pulses from PAL-XFEL.^28,29^ The benefits of using SXFEL are as follows: (1) the photoionization cross-sections for soft x-rays <1 keV are two orders of magnitude larger than those for hard x-rays >4 keV (Ref. 30) and (2) the bandpass of SXFEL is one order of magnitude smaller than that of XFEL. The measured *I* 3*d* XPD profiles for the aligned IPh molecules were compared with those via density functional theory (DFT) calculations. On the basis of the intensive comparison between these experimental and theoretical results, we propose the most favorable experimental conditions to realize the UXPD, which will be able to capture the transient structure of molecules during photochemical reactions. We also discuss specific applicability of UXPD for tracking photodissociation under the currently achievable experimental conditions.

## II. EXPERIMENT

### A. Experimental setup and procedure

**FIG. 1.**
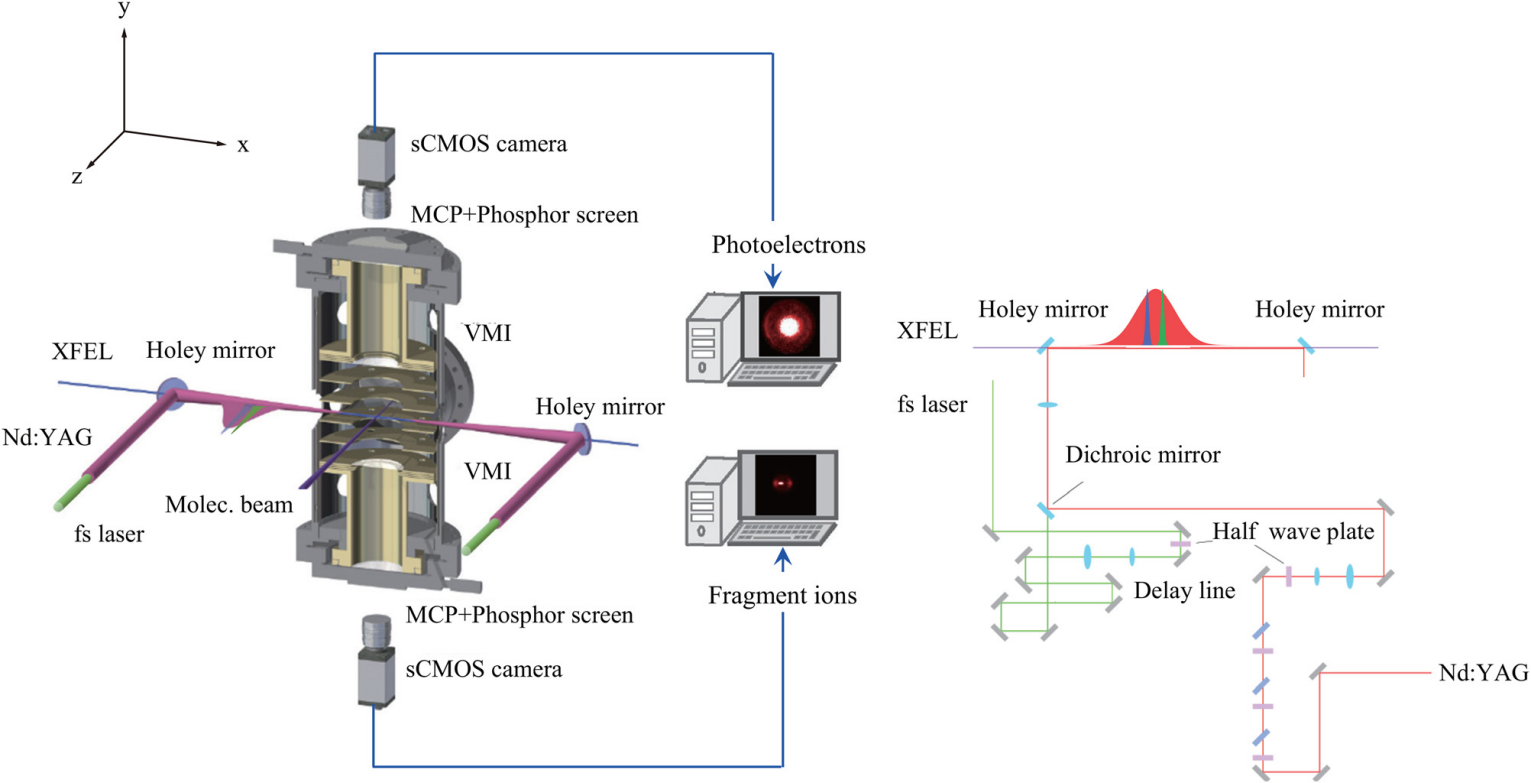
Schematic of the experimental setup (left) and optical paths of alignment, pump, and probe lasers (right). Laser beams propagating along the x-axis in a collinear arrangement intersect a supersonic pulsed molecular beam along the z-axis at the center of a vacuum chamber, but the pump laser was not used in this work. The Nd:YAG laser is used to adiabatically align the sample molecules that are probed by the SXFEL. XPD images of the photoelectrons are recorded by the upper VMI. The degree of alignment is quantified using the 2D momentum distributions of the fragment ions, which are registered by the lower VMI.

The experiments were conducted at the soft x-ray scattering and spectroscopy (SSS) beamline of PAL-XFEL.^31^ SXFEL pulses, with the energy of ∼100 *μ*J/pulse and duration of ∼50 fs, were fired into the interaction region of our diffractometer comprising the VMI spectrometer, which has been previously introduced in Refs. 25 and 26 and is schematically shown in Fig. 1. The typical bandwidth of an SXFEL is ∼0.5%, i.e., ∼4 eV at the photon energy of 750 eV. The injection-seeded Nd:YAG laser pulses (Spectra Physics, PRO 230–50), with the energy of ∼700 mJ/pulse and duration of ∼10 ns, were focused by a plano–convex lens placed outside the vacuum chamber. SXFEL and Nd:YAG laser beams were merged collinearly by a holey mirror inside the vacuum chamber. The spatial overlap of the SXFEL and Nd:YAG laser beams was first examined by monitoring images on a Ce:YAG phosphor screen in the interaction region, and then, the spatial overlap was confirmed by monitoring the degree of alignment of the sample molecules. The typical spot size of SXFEL was ∼60 and ∼80 *μ*m in the y- and z-directions for its 1/*e*^2^ values, respectively, as shown in Fig. 1. The spot size of the Nd:YAG laser is ∼60 *μ*m in the full width at half maximum with the Gaussian distribution. The temporal overlap between the SXFEL and Nd:YAG laser pulses was monitored using a fast photodiode. The polarization vectors of both the SXFEL and Nd:YAG laser beams were parallel to the z-direction.

Transistor-transistor logic (TTL) signals delivered from the PAL-XFEL facility, which fires XFEL pulses with the repetition rate of 30 Hz, were used as a master trigger for the present timing measurements. The master trigger was guided to the pulse generator (Quantum Composers, Model 9250) for the SXFEL pulses to synchronize with the Nd:YAG laser pulses, the pulse valve for molecular beam, and the shutter of the sCMOS cameras. The repetition rates were 30 Hz for both the Nd:YAG laser and sCMOS cameras and 15 Hz for the pulse valve.

A pulsed supersonic molecular beam was formed by expanding a gas mixture of the sample IPh molecules diluted in 40 bar helium through a pulsed valve, which is developed by Even and Lavie,^32^ into the vacuum chamber. The pulsed valve was heated to 60 °C to provide a partial pressure of ∼1 kPa for the sample IPh molecules. The molecular beam passed through a 3-mm-diameter skimmer, and it was introduced into the interaction region, where the Nd:YAG laser and SXFEL pulses overlapped. The source and main chambers were differentially pumped by turbo-molecular pumps, and their typical pressures during the experiments were 3 × 10^−4^ and 2.5 × 10^−6 ^Pa, respectively. The pulse duration of the valve was settled to 22 *μ*s by monitoring the pressure of the source chamber. The timing of the valve was optimized by observing the ion signals of the VMI.

The photoions and photoelectrons produced by the SXFEL pulses were measured using the faced VMIs.^25,26^ The velocity-focused photoelectrons were detected by a chevron-stacked dual microchannel plate (MCP) backed by a phosphor screen. The image on the phosphor screen was recorded shot by shot using the sCMOS camera, and it is stored on the PC. The photoelectron 2D momentum images obtained with and without the sample gas were alternately measured, and the objective images that originated from the residual-free sample gas were then obtained by subtracting the latter from the former. Both the photoelectron detection and the fragment photoions were accelerated simultaneously toward the other VMI, and they were detected by the same system. We acquired a total momentum image data of 50 000 and 20 000 shots for photoelectrons and fragment ions, respectively.

### B. Measured results

#### 1. Degree of alignment of iodobenzene molecules

**FIG. 2.**
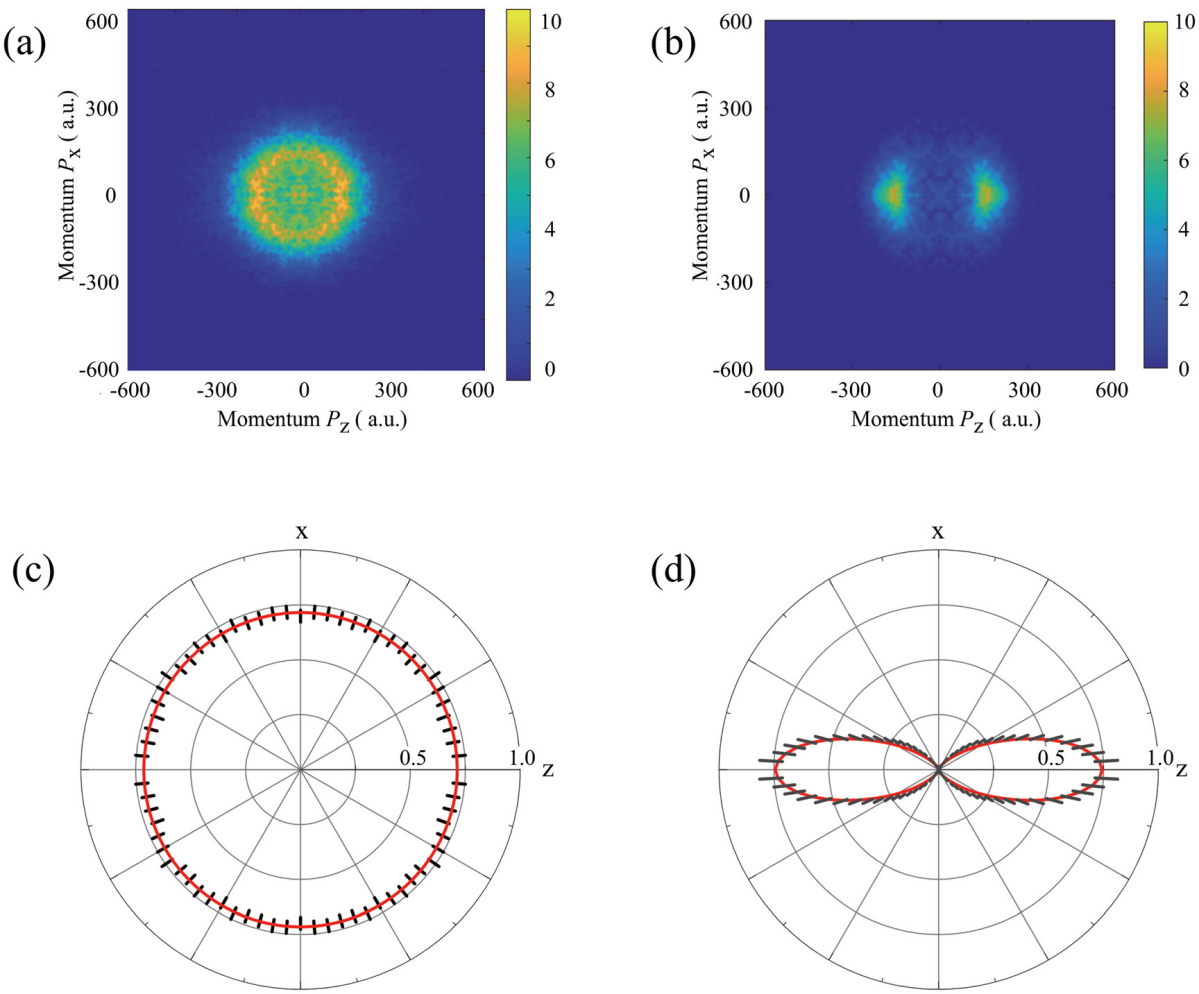
2D momentum images of fragment ion *I*^+^ without (a) and with (b) Nd:YAG laser pulses and their polar plots, (c) and (d). Fragment ion *I*^+^ image (b), reflecting the *C*–*I* axis distributions of IPh is aligned along the polarization vector of the Nd:YAG laser. The molecular axis distribution (d), expressed by the polar plot is described by Eq. (1), see text. In (c) and (d), the short bars denote the statistical errors of the experimental data, and the solid red curves are the fitting results. The polar plots in (c) and (d) were obtained from Abel-inverted images of the 2D images in (a) and (d). The degrees of alignment ${\left\langle {\text{cos}}^{2}\theta \right\rangle }_{1D}$ without and with Nd:YAG laser were evaluated from the polar plots (c) and (d), respectively, see text.

By absorbing an x-ray photon of 750 eV, IPh molecules are multiply ionized via Auger decays, and then, fragment photoions are produced by the Coulomb explosion. Among various fragment photoions, we selected *I*^+^ ions by applying the pulse-gate voltage to the MCP of the VMI because they dissociate along the *C*–*I* axis of the IPh molecules. Hence, on the basis of the measured angular distributions of *I*^+^ ions, we evaluated the degree of alignment for the polarization vector of the Nd:YAG laser. Figures 2(a) and 2(b) show the *I*^+^ ion images of IPh generated by SXFEL pulses without the alignment Nd:YAG laser and with the Nd:YAG laser, respectively. The images are presented in the laboratory frame of reference, whose z- and x-axis are the polarization vector of the Nd:YAG laser and its propagation direction, respectively, as shown in Fig. 1. In the IPh molecular ensembles, which are aligned adiabatically by the electric fields of the Nd:YAG laser, the “head vs tail” distinction is lost, and the distribution is axially symmetric for the *C*–*I* axis. That is, the angular distributions of *I*^+^ ions are described by only the mutual angle, Θ, between the *C*–*I* axis and the polarization vector of the Nd:YAG laser. Hence, the raw data in the quad screen of the image of upper and lower sides and those of left and right sides were averaged in the figures. With this average procedure, the position-dependent detection efficiencies of the MCP can be removed.

Without the alignment Nd:YAG laser, the *I*^+^ ions exhibit an isotropic distribution because the *C*–*I* axis of IPh is randomly oriented. Indeed, the degree of alignment, $\left\langle \;{\text{cos}}^{2}\Theta \right\rangle =0.33$, has been evaluated from the angular distribution of *I*^+^ ions shown in Fig. 2(c). With the alignment Nd:YAG laser, the angular distribution is confined along the z-axis, i.e., the *C*–*I* axis of IPh is aligned preferentially along the z-axis. To determine the degree of alignment of the IPh molecular ensembles, we simulated the angular distributions using the rotational temperature of molecules and the peak intensity of the Nd:YAG laser pulses, following *Evaluation of degree of molecular alignment* in the Methods section of Ref. 26. In the present case, as IPh is an asymmetry top rotor, the polarizability anisotropy of it is approximated by subtracting the average of the two lower components from the largest one, which is parallel to the *C*–*I* axis of IPh. Then, the simulation with the rotational temperature of 8 K and peak intensity of 7 × 10^11^ W/cm^2^ resulted in the angular distribution of

$$f(\Theta )=0.0197+0.0617P_{2}+0.0457P_{4}+0.0219P_{6}+0.009P_{8}+0.004P_{10}+0.002P_{12},$$ (1)

where *P_n_* is the *n*th Legendre polynomial. Equation (1) reproduces the measured one fairly well, as shown in Fig. 2(d). With this function, the expectation value of $\;{\text{cos}}^{2}\Theta$, i.e., the degree of alignment, has been evaluated:

$$\left\langle \;{\text{cos}}^{2}\Theta \right\rangle =0.78\pm 0.01.$$ (2)

#### 2. *I* 3*d* laboratory frame photoelectron angular distribution from aligned iodobenzene molecules

**FIG. 3.**
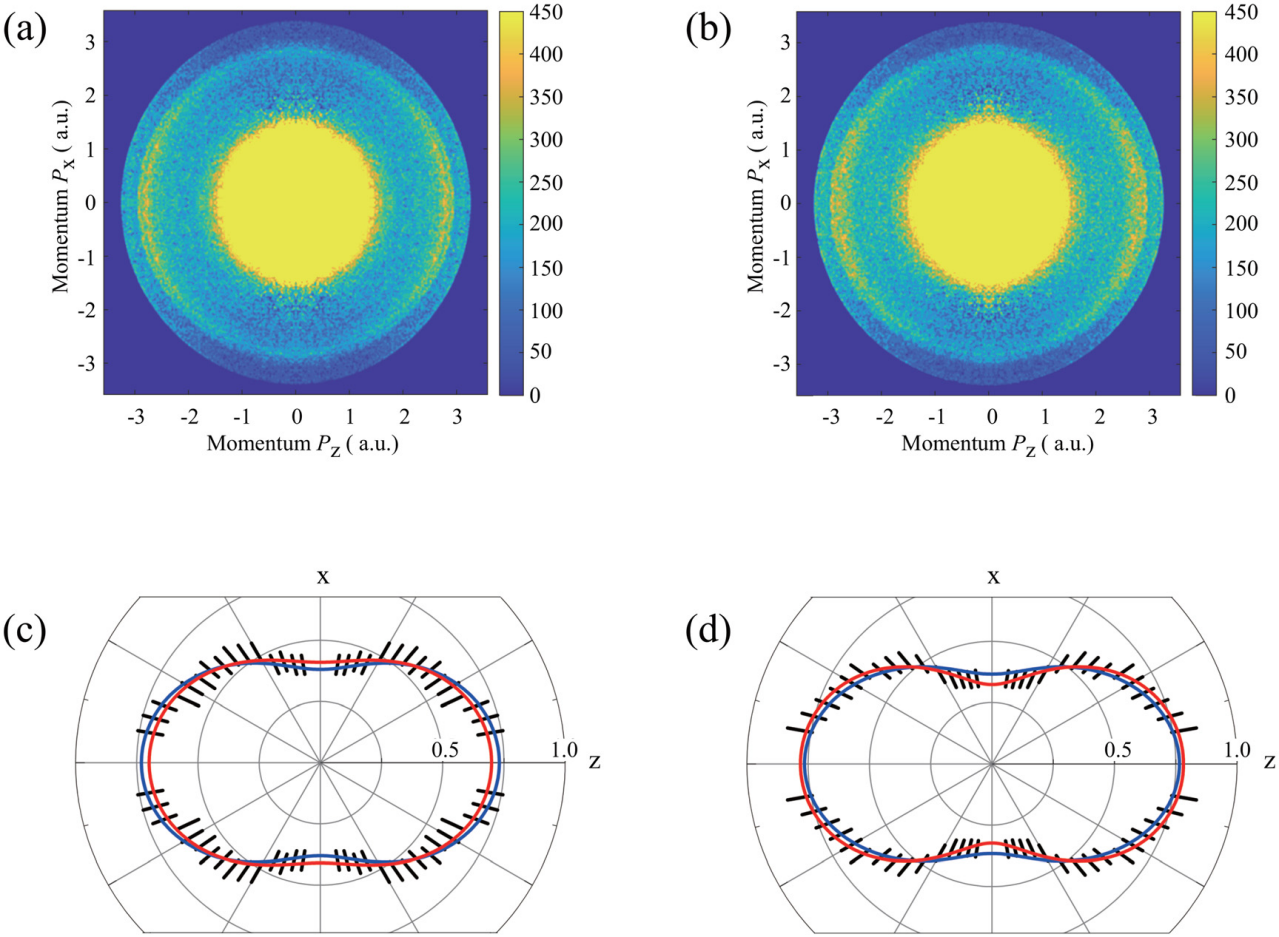
2D momentum images of *I* 3*d* photoelectrons from IPh without (a) and with (b) Nd:YAG laser pulses and their polar plots, (c) and (d). In (c) and (d), the short bars denote the statistical errors of the experimental data. The red curves are the fitted results, and the blue curves the calculated. Both results are normalized by the integrated cross sections. The *I* 3*d* photoelectron angular distribution (c) in the laboratory frame without Nd:YAG laser, i.e., ${\text{LFPAD}}_{\text{random}}^{3d}$, is written by the asymmetry parameter *β*; ${\text{LFPAD}}_{\text{random}}^{3d}(\theta^{\prime })=\frac{\sigma }{4\pi }\left(1+\beta P_{2}(\text{cos}\;\theta^{\prime })\right)$. The experimental *β*-value of 0.38 ± 0.014 was obtained by the fitting, and the calculated *β*-value was 0.47, see text.

Figures 3(a) and 3(b) show 2D *I* 3*d* photoelectron images of IPh without and with the Nd:YAG laser, respectively, in the laboratory frame. These images were measured with SXFEL pulses with a photon energy of 750 eV. In the figures, the raw data in the quad screen of the image of upper and lower sides and those of left and right sides were averaged because of the symmetry restriction. The inner and outer rings around the radius of 2.5 a.u. correspond to *I* 3*d*_3/2_ and *I* 3*d*_5/2_ photoelectrons, respectively. Although the degrees of alignment of the sample IPh molecular ensembles are quite different, as shown in Fig. 2, the differences between the *I* 3*d* photoelectron images from the randomly oriented ensembles and that from the aligned molecular ensembles are barely discernible. To discuss more details about the differences, the polar plots relevant to Figs. 3(a) and 3(b) with the current theoretical results are shown in Figs. 3(c) and 3(d), respectively. The polar plots were obtained from the outer ring image since we confirmed that there are no detectable differences between the polar plots from the outer and inner ring images. Compared with *I* 3*d* laboratory frame photoelectron angular distribution (LFPAD) from the randomly oriented IPh (hereafter ${\text{LFPAD}}_{\text{random}}^{3d}$), *I* 3*d* laboratory frame photoelectron angular distribution from the aligned IPh (hereafter ${\text{LFPAD}}_{\text{align}}^{3d}$) exhibits a slightly preferential direction along the polarization vectors of both the alignment Nd:YAG laser and XFEL, as shown in Figs. 3(c) and 3(d). Both the comparison between the experiment and theory and the reason why the ${\text{LFPAD}}_{\text{align}}^{3d}$ has such a structureless simple profile are explained in Sec. IV B.

**FIG. 4.**
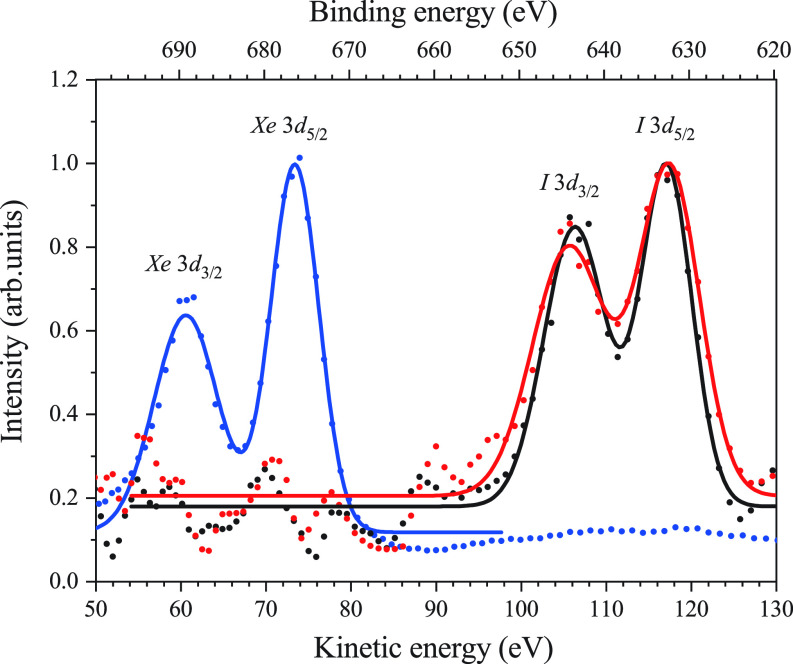
3*d* photoelectron spectra of *Xe* and *I* of IPh. *Xe* (blue line) and IPh (red and black lines) spectrum were measured by different runs, but under the same conditions of the VMI. Thus, three spectra are overwritten in the figure for comparison. Red and black line express the photoelectron spectra of IPh with and without Nd:YAG laser, respectively. Vogit functions fitted to the experimental data are depicted.

The photoelectron spectra obtained from Figs. 3(a) and 3(b) are shown in Fig. 4, where the spin–orbit splitting of *I* 3*d* sub-shells is resolved. The 3*d* photoelectron spectrum of *Xe*, which was reported in Ref. 33, is also depicted in Fig. 4 as a reference since the photoelectrons of IPh were measured under the same VMI conditions and photon energy as those for *Xe*. Therefore, referring to the ionization potentials (IPs) of *Xe* 3*d*_5/2,3/2_ in Ref. 34, we determined the IPs of IPh: 632.64 ± 0.8 and 643.41 ± 0.8 eV for the *I* 3*d*_5/2_ and *I* 3*d*_3/2_, respectively. On the one hand, the relativistic calculations with Grasp92 + RATIP^35^ gave the values of 628.540 and 639.938 eV for j = 2 *I* 3*d*_5/2_5*p*_3/2_ and j = 2 *I* 3*d*_3/2_5*p*_3/2_, respectively.

As shown in Fig. 4, the spectra exhibit the different spectral widths. The bandwidth of 8.31 eV for IPh is broader than that of 6.90 eV for *Xe* due to the vibrational broadening in the IPh molecules. Furthermore, there is a slight change of spectral widths, i.e., from 8.31 to 10.09 eV for *I* 3*d*_5/2_ without and with the Nd:YAG lasers. This broadening in the IPh molecules is due to the above-threshold ionization (ATI) induced by the Nd:YAG laser fields. We have estimated less than three sidebands due to ATI under the present experimental conditions, referring to Ref. 36. On the other hand, the ponderomotive energy is estimated as 0.074 eV from the Nd:YAG laser intensity of 0.7 TW/cm^2^.^36^ On the basis of these considerations, we think that the angular distributions of the *I* 3*d* photoelectrons are unaffected by the electric fields of the Nd:YAG laser.

## III. FUNCTIONAL FORM OF PHOTOELECTRON ANGULAR DISTRIBUTION FROM ALIGNED MOLECULES

### A. Molecular frame photoelectron angular distribution

**FIG. 5.**
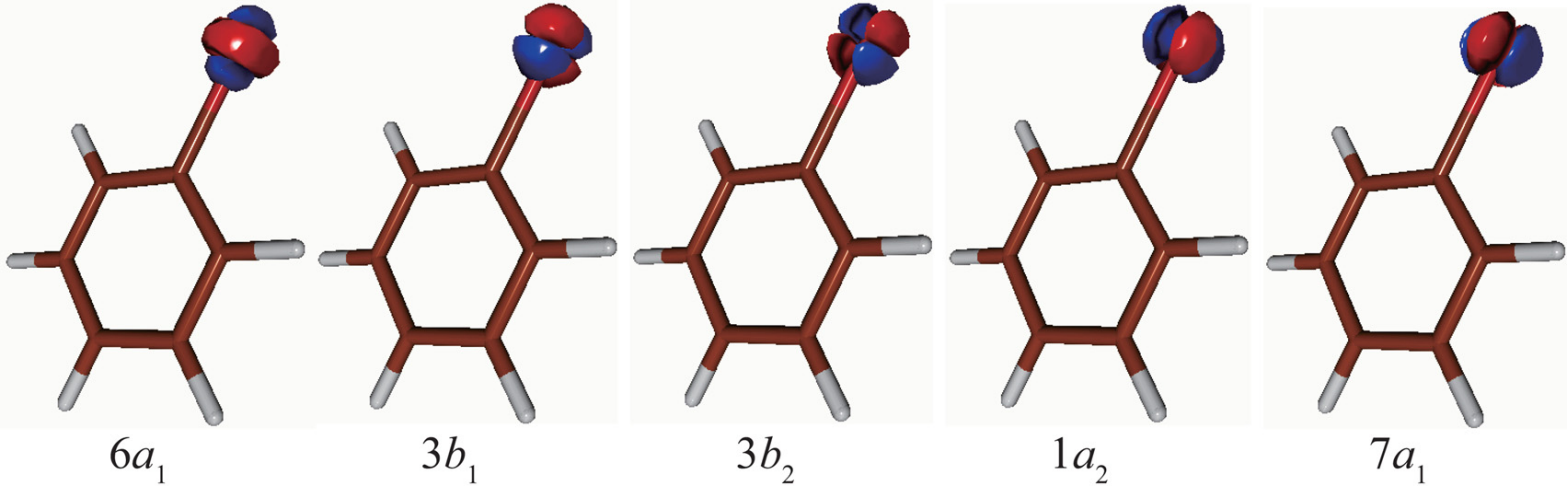
Molecular orbitals of IPh molecule related to iodine 3*d* atomic orbitals in the non-relativistic picture.

The ground state electronic density of IPh and the continuum wave functions have been calculated with the nonrelativistic DFT, thereby employing the LB94 exchange correlation functional.^37^ On the basis of the electron diffraction and microwave spectroscopy data, the equilibrium molecular structure of IPh was determined.^38^ IPh belongs to the *C*_2__*v*_ point group; hence, the nomenclature of the molecular orbitals is given by the conventional definition in the molecular frame (MF) of reference, where the Z-axis represents the principal symmetry axis of the *C*–*I* and the Y-axis is orthogonal to the Z-axis and on the plane of the phenyl ring. In the nomenclature for *C*_2__*v*_, the iodine 3*d* atomic orbitals ( $3d_{z^{2}}$, $3d_{xz}$, $3d_{yz}$, $3d_{x^{2}-y^{2}}$, and $3d_{xy}$) of IPh are related to the 6*a*_1_, 3*b*_1_, 3*b*_2_, 1*a*_2_, and 7*a*_1_ lone-pair molecular orbitals, respectively, as shown in Fig. 5. The initial electron density is taken from a conventional bound-state LCAO-DFT calculation, with the program Amsterdam Density Functional (ADF).^39^ The computations have then been performed, applying a basis set of multicenter B-spline functions that are centered on all the nuclei.^40,41^ The origin is placed on the *C*1 nucleus of IPh. The maximum angular momentum of the spherical wave expansion of the continuum is chosen as *l*_max_ = 18. The order of B-splines is 10. A linear radial grid with a step size *h *=* *0.25 bohr, up to the maximum of 25.00 bohr, is applied for the one-center expansion. The maximum angular momentum applied in the off-center expansion has been set to 2. The maximum radii for the off-center expansion are fixed at 1.8, 1.0, and 0.8 bohr for the iodine, carbon, and hydrogen atoms, respectively. The photoelectron kinetic energy has been set to 120 eV, considering the photon energy of 750 eV and the experimentally determined IP of 632.64 eV for the *I* 3*d*_5/2_ of IPh. All the computations apart from running ADF have benefited from the DTU Computing Center resources.^42^

MFPADs are described by the dipole transition moments as follows:

$$D_{lh}^{p\mu -}(m_{\gamma })=\left\langle \varphi_{\mathrm{klh}}^{p\mu -}\right|rY_{1m_{\gamma }}\left|\varphi_{i}\right\rangle ,$$ (3)

where $\varphi_{i}$ is the Kohn–Sham orbital that is currently ionized and $\varphi_{\mathrm{klh}}^{p\mu -}$ is the continuum orbitals normalized to incoming wave **S**-matrix boundary conditions. In $\varphi_{\mathrm{klh}}^{p\mu -}$, *p* is the irreducible representation, *μ* is its subspecies in the case of degeneracy, *l* is the angular momentum, and *h* is an index that identifies independent contribution when *p* and *l* are the same. These objects are related to the unitary transformation of spherical harmonics (*Y_lm_*) into real symmetry adapted spherical harmonics ( $X_{lh}^{p\mu }$),^43^

$$X_{lh}^{p\mu }(\theta ,\phi )=\sum_{m}Y_{lm}(\theta ,\phi )b_{\mathrm{lmh}}^{p\mu }.$$ (4)

The functional form of MFPAD is expressed by the general treatment of Refs. 43 and 44:

$$\frac{d^{2}\sigma }{d\Omega d\hat{k}}={\left(-1\right)}^{m_{p}}4\pi^{2}\alpha \omega n_{i}\left(\frac{4\pi }{3}\right)\sum_{L}\sum_{M\prime =-L}^{L}A_{LM\prime }(k,\Omega )Y_{LM\prime }(\hat{k}),$$ (5)

where Ω = (Φ, Θ, Χ) represents the Euler angles that define the lab frame (LF) with respect to the molecular frame (MF), $\hat{k}=(\theta ,\phi )$ expresses the direction of the photoelectron momentum in the MF, *α* is the fine structure constant, *ω* is the incident photon energy, *n_i_* is the occupation number of the ionized orbital, and *m_p_* is the polarization quantum number: 0 and +1 or −1 for linear and left or right circular polarization, respectively. The *A_LM′_* coefficients, which appear in Eq. (5), are calculated from dipole matrix elements and phase shifts as follows:

$$\begin{array}{c} A_{LM\prime }(k,\Omega )=\sum_{\begin{array}{c} p\mu \mathrm{hlm}m_{\gamma }\\ p\prime \mu \prime h\prime l\prime m\prime m\prime_{\gamma } \end{array}}{\left(-i\right)}^{l-l^{\prime }}e^{i\left(\sigma_{l}-\sigma_{l^{\prime }}\right)}{\left(-1\right)}^{m+m_{\gamma }}{\left(\frac{\left(2l+1\right)\left(2l^{\prime }+1\right)\left(2L+1\right)}{4\pi }\right)}^{1/2}\\ \times \left(\begin{array}{c} \begin{array}{ccc} l & l^{\prime } & L \end{array}\\ \begin{array}{ccc} 0 & 0 & 0 \end{array} \end{array}\right)\left(\begin{array}{ccc} l & l^{\prime } & L\\ -m & m^{\prime } & M^{\prime } \end{array}\right)b_{\mathrm{lmh}}^{p\mu }b_{l\prime m\prime h\prime }^{p^{\prime }\mu^{\prime }*}D_{lh}^{p\mu -}(m_{\gamma })D_{l\prime h\prime }^{p\prime \mu^{\prime }-}({m^{\prime }}_{\gamma })*\\ \times \sum_{JM_{J}^{\prime }}\left(2J+1\right)\left(\begin{array}{ccc} 1 & 1 & J\\ -m_{p} & m_{p} & 0 \end{array}\right)\left(\begin{array}{ccc} 1 & 1 & J\\ -m_{\gamma } & m_{\gamma }^{\prime } & M_{J}^{\prime } \end{array}\right)R_{M_{J}^{\prime },0}^{J}(\Omega ), \end{array}$$ (6)

where $R_{M_{J}^{\prime },0}^{J}(\Omega )$ is the rotation matrices, $MF\;(X,Y,Z)\overset{R}{\to }LF\;(x,y,z)$, $\sigma_{l}$ is the Coulomb phase shifts, and the Wigner 3*j* symbols are applied.

### B. Laboratory frame photoelectron angular distribution from aligned molecules

The spherical harmonics $Y_{LM\prime }(\hat{k})$ in the MF are expressed by the relevant function $Y_{LM}({\hat{k}}^{\prime })$ in the LF:

$$Y_{LM\prime }(\hat{k})=\sum_{M}Y_{LM}({\hat{k}}^{\prime })R_{M\prime M}^{L*}(\Omega ).$$ (7)

By substituting Eq. (7) into Eq. (5) and then the following formulas of the product of rotation matrices, we have

$$R_{M^{\prime }M}^{L*}(\Omega )R_{M_{J}^{\prime },0}^{J}(\Omega )={\left(-1\right)}^{M_{J}^{\prime }}\sum_{\mathrm{KQQ}\prime }\left(2K+1\right)\left(\begin{array}{ccc} L & J & K\\ M\prime & -M_{J}\prime & Q\prime \end{array}\right)\left(\begin{array}{ccc} L & J & K\\ M & 0 & Q \end{array}\right)R_{Q^{\prime }Q}^{K}(\Omega ).$$ (8)

The MFPAD of Eq. (5) is rewritten in the LF as follows:

$$\frac{d^{2}\sigma }{d\Omega d{\hat{k}}^{\prime }}={\left(-1\right)}^{m_{p}}4\pi^{2}\alpha \omega n_{i}\left(\frac{4\pi }{3}\right)\sum_{L}\sum_{M=-L}^{L}{\bar{A}}_{LM}(k,\Omega )Y_{LM}({\hat{k}}^{\prime }),$$ (9)

where

$$\begin{array}{c} {\bar{A}}_{LM}(k,\Omega )=\sum_{\begin{array}{c} p\mu \mathrm{hlm}m_{\gamma }\\ p\prime \mu \prime h\prime l\prime m\prime m\prime_{\gamma } \end{array}}{\left(-i\right)}^{l-l^{\prime }}e^{i\left(\sigma_{l}-\sigma_{l^{\prime }}\right)}{\left(-1\right)}^{m-m_{\gamma }^{\prime }}{\left(\frac{\left(2l+1\right)\left(2l\prime +1\right)\left(2L+1\right)}{4\pi }\right)}^{1/2}\\ \times \sum_{M\prime }\left(\begin{array}{c} \begin{array}{ccc} l & l\prime & L \end{array}\\ \begin{array}{ccc} 0 & 0 & 0 \end{array} \end{array}\right)\left(\begin{array}{ccc} l & l^{\prime } & L\\ -m & m^{\prime } & M\prime \end{array}\right)b_{\mathrm{lmh}}^{p\mu }b_{l\prime m\prime h\prime }^{p\prime \mu \prime *}D_{lh}^{p\mu -}(m_{\gamma })D_{l\prime h\prime }^{p\prime \mu \prime -}(m\prime_{\gamma })*\\ \times \sum_{JM_{J}\prime KQ}\left(2J+1\right)\left(2K+1\right)\left(\begin{array}{ccc} 1 & 1 & J\\ -m_{p} & m_{p} & 0 \end{array}\right)\left(\begin{array}{ccc} 1 & 1 & J\\ -m_{\gamma } & {m^{\prime }}_{\gamma } & M_{J}\prime \end{array}\right)\times \left(\begin{array}{ccc} L & J & K\\ M\prime & -M\prime_{J} & Q \end{array}\right)\left(\begin{array}{ccc} L & J & K\\ M & 0 & -M \end{array}\right)R_{Q,-M}^{K}(\Omega ). \end{array}$$ (10)

Here, we consider the molecular-axis distributions of the aligned molecular ensemble, which have been mentioned in Sec. II B 1. Such distributions are expressed generally as follows:^45^

$$f(\Omega^{-1})=\sum_{K=\text{even}}F_{K}P_{K}(\text{cos}\;(-\Theta )).$$ (11)

Therefore, once Eq. (10) has been multiplied by the distribution function of Eq. (11) and then integrated over Ω, applying the relation between the rotational matrix and the Legendre polynomial $P_{K}(\text{cos}\;(-\Theta ))=R_{00}^{K*}(\Omega^{-1})$ and the orthogonality of the rotational matrices, ${\text{LFPAD}}_{\text{align}}^{3d}$ considering the distribution is written as follows:

$$\frac{d\sigma }{d{\hat{k}}^{\prime }}={\left(-1\right)}^{m_{p}}4\pi^{2}\alpha \omega n_{i}\left(\frac{4\pi }{3}\right)\sum_{L=\text{even}}\sum_{M=-L}^{L}\left\langle A_{L}\right\rangle P_{L}(\text{cos}\;\theta \prime ),$$ (12)

where the expansion coefficient $\left\langle A_{L}\right\rangle$ is given by

$$\begin{array}{c} \left\langle A_{L}\right\rangle =\frac{2L+1}{4\pi }\sum_{\begin{array}{c} p\mu \mathrm{hlm}m_{\gamma }\\ p\prime \mu \prime h\prime l\prime m\prime m\prime_{\gamma } \end{array}}\sum_{JM_{J}K}{\left(-i\right)}^{l-l^{\prime }}e^{i\left(\sigma_{l}-\sigma_{l^{\prime }}\right)}{\left(-1\right)}^{m^{\prime }+m_{\gamma }}{\left(\left(2l+1\right)\left(2l^{\prime }+1\right)\right)}^{1/2}\times \left(\begin{array}{c} \begin{array}{ccc} l & l\prime & L \end{array}\\ \begin{array}{ccc} 0 & 0 & 0 \end{array} \end{array}\right)\left(\begin{array}{ccc} l & l^{\prime } & L\\ -m & m^{\prime } & M_{J} \end{array}\right)b_{\mathrm{lmh}}^{p\mu }b_{l\prime m\prime h\prime }^{p\prime \mu \prime *}D_{lh}^{p\mu -}(m_{\gamma })D_{l\prime h\prime }^{p\prime \mu \prime -}{\left(m\prime_{\gamma }\right)}^{*}\\ \times \left(2J+1\right)\left(\begin{array}{ccc} 1 & 1 & J\\ -m_{p} & m_{p} & M_{J} \end{array}\right)\left(\begin{array}{ccc} 1 & 1 & J\\ -m_{\gamma } & {m^{\prime }}_{\gamma } & 0 \end{array}\right)\\ \times \left(\begin{array}{ccc} L & J & K\\ M_{J} & -M_{J} & 0 \end{array}\right)\left(\begin{array}{ccc} L & J & K\\ 0 & 0 & 0 \end{array}\right)F_{K}, \end{array}$$ (13)

and $\theta^{\prime }$ stands for the polar angle of the photoelectron momentum in the LF.^46,47^

For the aligned molecular ensembles, Ω = (Φ, Θ, Χ) reduces to Ω = (0, Θ, 0); thus, ${\text{LFPAD}}_{\text{align}}^{3d}$ depends on the two parameters of $\left(\Theta \;;\;\;\theta^{\prime }\right)$. When the polar angle Θ is fixed at a certain value $\Theta_{0}$, the distribution function is expressed by the delta function of

$$\delta (\text{cos}\;\Theta -\text{cos}\;\Theta_{0})=\sum_{K}\frac{2K+1}{2}P_{K}(\text{cos}\;\Theta_{0})P_{K}(\text{cos}\;\Theta ),$$ (14)

and ${\text{LFPAD}}_{\Theta_{0}}^{3d}(\;\theta^{\prime })$ is written by

$${\text{LFPAD}}_{\Theta_{0}}^{3d}(\theta^{\prime })=\int {\text{LFPAD}}_{\text{align}}^{3d}(\Theta ;\theta^{\prime })\delta (\text{cos}\;\Theta -\text{cos}\;\Theta_{0})\;\text{sin}\;\Theta d\Theta .$$ (15)

The functional form ${\text{LFPAD}}_{\Theta_{0}}^{3d}(\theta^{\prime })$ can be obtained by inserting $F_{K}=\left(2K+1/2\right)P_{K}(\text{cos}\;\Theta_{0})$ into Eq. (13).

## IV. COMPUTED RESULTS

### A. MFPAD

As mentioned in the introduction, the MFPAD for the inner-shell photoelectrons having energies of >100 eV can be interpreted as an XPD profile. The XPD profile is formed by the direct photoemission wave, the singly scattered wave, and the interference between them. The molecular structure is reflected in the XPD profile mainly through the interference terms, which are inversely proportional to the internuclear distances.^48^ Therefore, the MFPAD reflects the molecular geometry, when the x-ray is polarized in the direction from the electron emitter to the scattering site, and the molecular orbital, which is ionized, has large distribution in this direction.

**FIG. 6.**
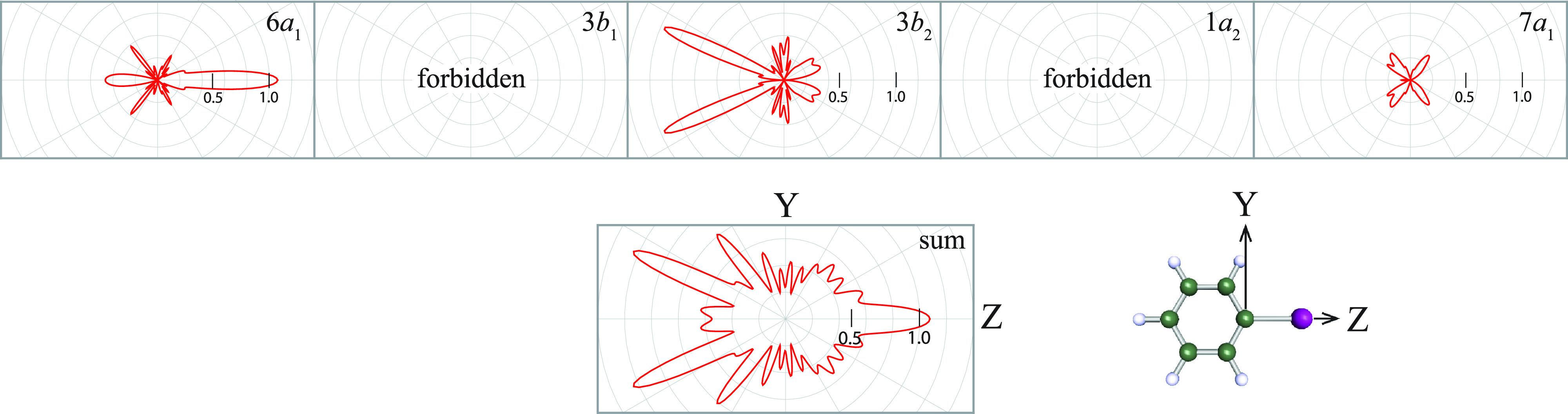
MFPADs from the 6*a*_1_, 3*b*_1_, 3*b*_2_, 1*a*_2_, and 7*a*_1_ orbitals of IPh. SXFEL polarization vector is parallel to the *C*–*I* axis, and the photoelectron energy is set to 120 eV. Panel below shows the sum of the five MFPADs with the geometry depicted by boll and stick molecular model of IPh.

In Fig. 6, the MFPADs from the nearly degenerate 6*a*_1_, 3*b*_1_, 3*b*_2_, 1*a*_2_, and 7*a*_1_ orbitals of IPh are shown on the YZ plane. On this plane, the polarization vectors of both the SXFEL and Nd:YAG laser are parallel to the *C*–*I* axis. However, on this plane, the MFPADs from the 1*a*_2_ and 3*b*_1_ orbitals are 0 because both the orbitals have nodes on the plane, as shown in Fig. 5. The scales of the plots reflect the relative values of the differential cross-sections from the 6*a*_1_, 7*a*_1_, and 3*b*_2_ orbitals and their sum. The shape of the MFPAD from the 6*a*_1_ orbital is basically like $f_{Z^{3}}$ wave, although the MFPAD exhibits the backward-scattering effect by the phenyl ring. The MFPAD from the 7*a*_1_ orbital barely exhibits a scattering effect because the 7*a*_1_ orbital is strongly distributed in the direction perpendicular to the *C*–*I* axis, as shown in Fig. 5. The MFPAD from the 3*b*_2_ orbital exhibits the forward-focusing effect^49^ by the phenyl ring because the 3*b*_2_ orbital has a relatively large distribution in the direction toward the *C*2 and *C*6 atoms. Moreover, the sum of MFPADs is characterized by the backward-scattering and forward-focusing effects by the phenyl ring.

### B. LFPAD from aligned iodobenzene

The calculated total ionization cross sections *σ* from the 6*a*_1_, 3*b*_1_, 3*b*_2_, 1*a*_2_, and 7*a*_1_ orbitals are 0.5206, 0.5264, 0.5247, 0.5221, and 0.5224 MB, respectively. The calculated asymmetry parameter *β* for the relevant five orbitals is 0.2665, 0.5995, 0.4234, 0.5419, and 0.5217, respectively. Hence, the calculated total *β* parameter for the *I* 3*d* orbitals of IPh is 0.47, which expresses the profile of ${\text{LFPAD}}_{\text{random}}^{3d}$. Although the experimental *β* parameter is 0.38 ± 0.014, the experimental and theoretical profiles of ${\text{LFPAD}}_{\text{random}}^{3d}$ are similar to each other, as shown in Fig. 3(c).

**FIG. 7.**
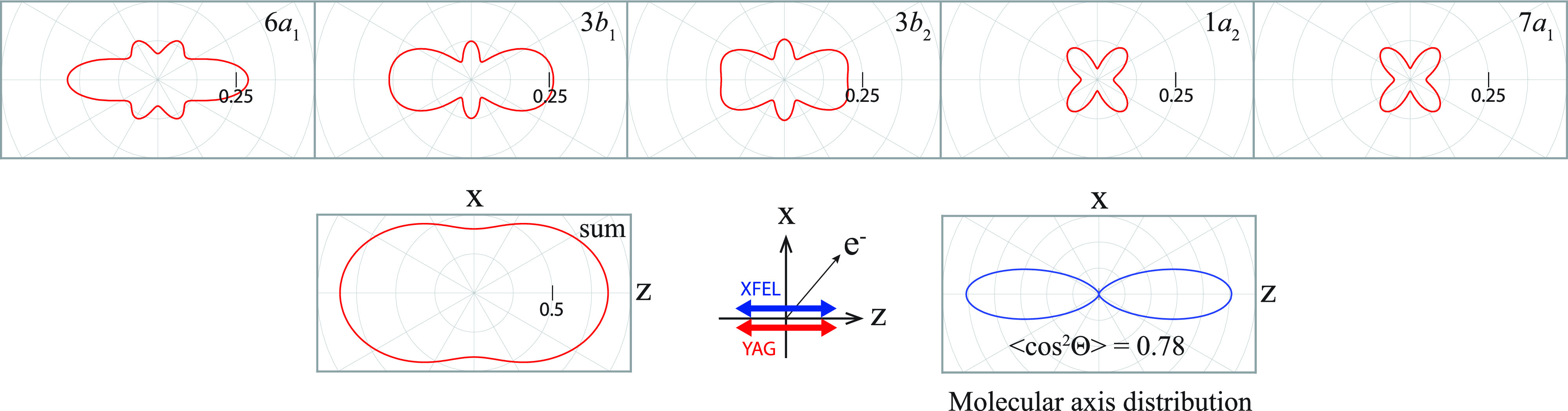
${\text{LFPAD}}_{\text{align}}^{p}$ from the 6*a*_1_, 3*b*_1_, 7*a*_1_, 3*b*_2_, 1*a*_2_, and 7*a*_1_ orbitals of the aligned molecular ensemble of IPh. The photoelectron energy was set to 120 eV. In the lower panel, left: the sum of the five ${\text{LFPAD}}_{\text{align}}^{p}$, middle: the polarization geometry, and right: the polar plot of the molecular axis distribution determined experimentally, the same as Fig. 2(d), which was used in the LFPAD calculations.

Figure 7 shows the calculated ${\text{LFPAD}}_{\text{align}}^{3d}$ on the zx plane from the nearly degenerate 6*a*_1_, 3*b*_1_, 3*b*_2_, 1*a*_2_, and 7*a*_1_ orbitals of IPh (hereafter ${\text{LFPAD}}_{\text{align}}^{p}$, where *p *=* *6*a*_1_, 3*b*_1_, …) and their sum (hereafter ${\text{LFPAD}}_{\text{align}}^{\text{sum}}$). In contrast to MFPAD, ionizations from both the 3*b*_1_ and 1*a*_2_ orbitals contribute to the ${\text{LFPAD}}_{\text{align}}^{3d}$. In the calculations, we used the experimentally determined value of the degree of alignment for the IPh molecules, $\left\langle {\text{cos}}^{2}\Theta \right\rangle =0.78$, where the values of *F_K_* substituted in Eq. (13) are the coefficients in Eq. (1). The measured ${\text{LFPAD}}_{\text{align}}^{3d}$ was compared with the calculated one, as shown in Fig. 3. Furthermore, in Fig. 3, the calculated result agrees with the measured one within the experimental uncertainties. The above-mentioned agreements of the *β* parameter and ${\text{LFPAD}}_{\text{align}}^{3d}$ between the theory and the experiment guarantee reliability of the present calculations.

To see the details of ${\text{LFPAD}}_{\text{align}}^{\text{sum}}$, each contribution from the nearly degenerate *I* 3*d* orbitals is examined by referring to Fig. 7. The backward-scattering effect in ${\text{LFPAD}}_{\text{align}}^{6a_{1}}$ is unclear, and the scattering effects in ${\text{LFPAD}}_{\text{align}}^{1a_{2}}$ and ${\text{LFPAD}}_{\text{align}}^{7a_{1}}$ are barely discernible because both the molecular orbitals do not have large density distributions in directions toward the phenyl ring (see Fig. 5). By contrast, the forward-focusing effects by the phenyl ring can still be observed in ${\text{LFPAD}}_{\text{align}}^{3b_{1}}$ and ${\text{LFPAD}}_{\text{align}}^{3b_{2}}$. Once the phenyl ring has tilted from the zx plane, the 3*b*_1_ and 3*b*_2_ orbitals (Fig. 5) have similar density distributions on the zx plane, and scattering effects by the phenyl ring on electrons emitted from these orbitals lead to similar LFPAD patterns. However, once the ${\text{LFPAD}}_{\text{align}}^{p}$ from the nearly degenerate five orbitals has been summed (see ${\text{LFPAD}}_{\text{align}}^{\text{sum}}$), the fine structures in the MFPAD, such as the backward-scattering and forward-focusing effects, are almost smeared out. Moreover, the calculated ${\text{LFPAD}}_{\text{align}}^{\text{sum}}$ hardly varies, even if the *C*–*I* bond length is changed by ±0.2 Å in the calculations. On the basis of these computed results, we can conclude that the degeneracies of the initial state eliminate the sensitivity on molecular structure in the ${\text{LFPAD}}_{\text{align}}^{\text{sum}}$.

**FIG. 8.**
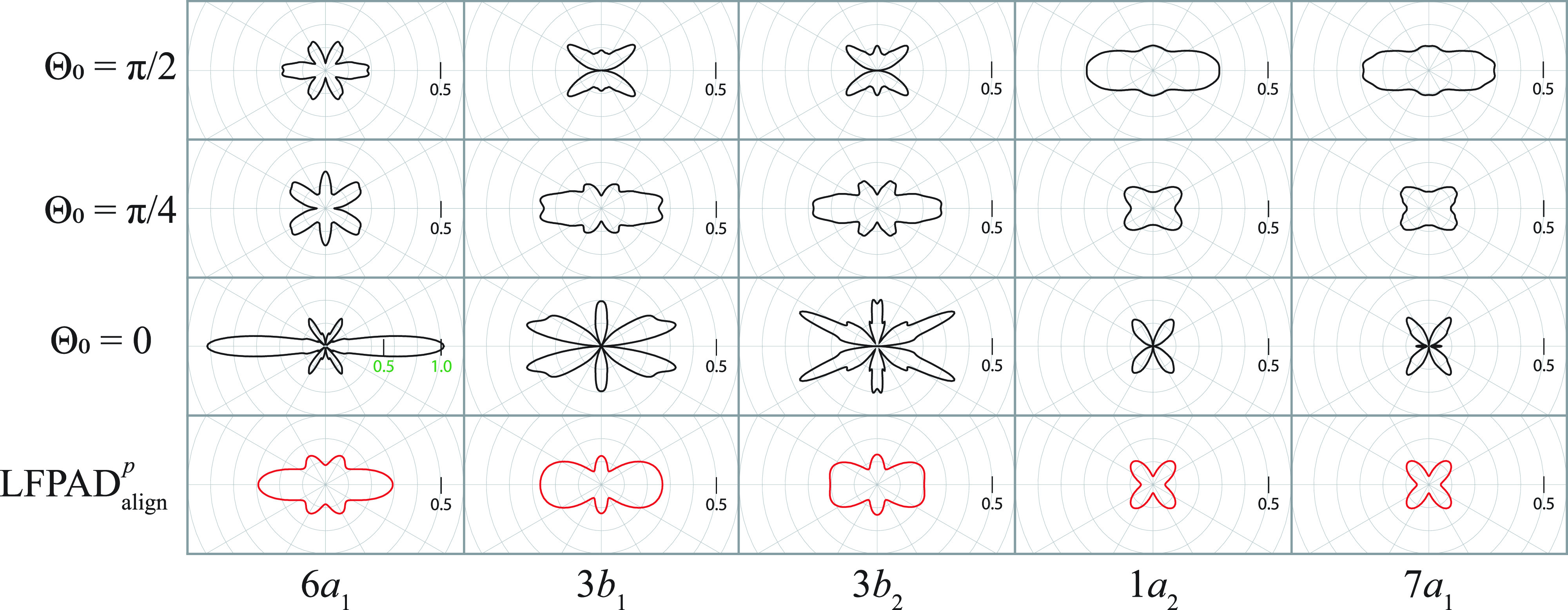
LFPADs depending on both the ionized molecular orbitals and the molecular alignment. The first, second, and third row show ${\text{LFPAD}}_{\Theta_{0}}^{p}(\theta^{\prime })$ at Θ_0_ = π/2, π/4, and 0, respectively, and the fourth row shows ${\text{LFPAD}}_{\text{align}}^{p}$ with the degree of alignment of $\left\langle \;{\text{cos}}^{2}\Theta \right\rangle =0.78$ that is the same as upper of Fig. 7. Both the molecular axis distribution and the delta function given by Eq. (14) are normalized with respect to integral over Θ. Note that the ${\text{LFPAD}}_{\Theta_{0}=0}^{6a_{1}}(\theta^{\prime })$ is plotted in a different scale.

To examine the possibility of observing the scattering effects in the ${\text{LFPAD}}_{\text{align}}^{\text{sum}}$, we calculated the ${\text{LFPAD}}_{\Theta_{0}}^{p}$ at $\Theta_{0}=\pi /2,\;\;\pi /4,\;\;\text{and}\;\;0$. The computed results of ${\text{LFPAD}}_{\Theta_{0}}^{p}$ for the five nearly degenerate *I* 3*d* orbitals are shown in Fig. 8. In the ${\text{LFPAD}}_{\Theta_{0}}^{p}$ at $\Theta_{0}=0$, the fine structures are formed by the backward-scattering and the forward-focusing effects, whereas the scattering effects are unobvious in the ${\text{LFPAD}}_{\Theta_{0}}^{p}$ at $\Theta_{0}=\pi /4\;\;\text{and}\;\;\pi /2$. Obviously, the fine structures formed by the scattering effects in the ${\text{LFPAD}}_{\Theta_{0}}^{6a_{1}}$, ${\text{LFPAD}}_{\Theta_{0}}^{3b_{1}}$, and ${\text{LFPAD}}_{\Theta_{0}}^{3b_{2}}$ at $\Theta_{0}=0$ are smeared out by the contributions of ${\text{LFPAD}}_{\Theta_{0}}^{p}$ at $\Theta_{0}=\pi /4\;\;\text{and}\;\;\pi /2$.

**FIG. 9.**
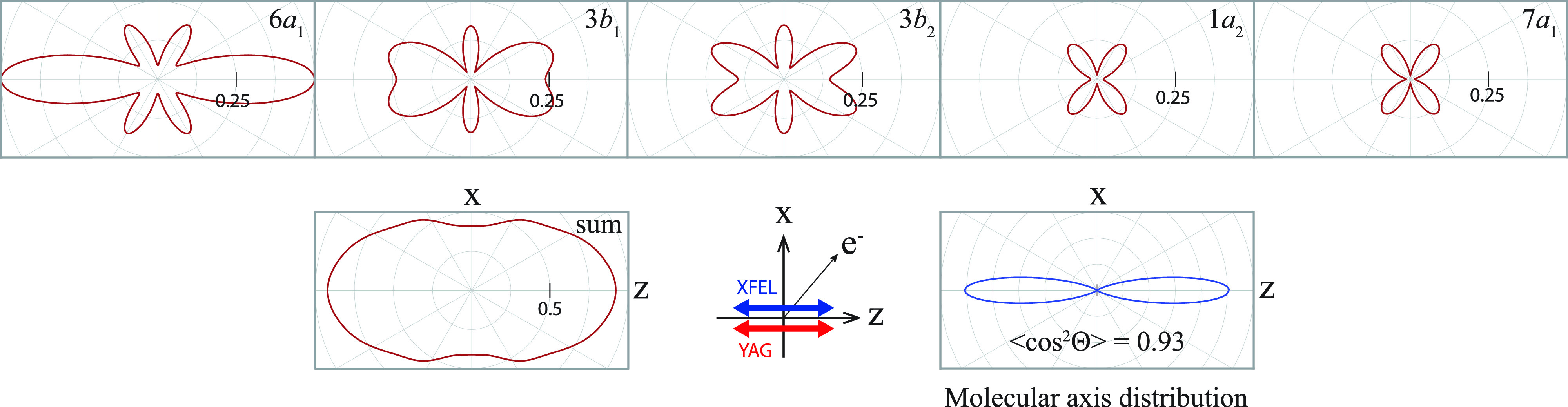
${\text{LFPAD}}_{\text{align}}^{p}$ from the 6*a*_1_, 3*b*_1_, 7*a*_1_, 3*b*_2_, 1*a*_2_, and 7*a*_1_ orbitals of the aligned molecular ensemble of IPh. The photoelectron energy was set to 120 eV, and the degree of alignment, $\left\langle \;{\text{cos}}^{2}\Theta \right\rangle =0.93$, was assumed. In the lower panel, left: the sum of the five ${\text{LFPAD}}_{\text{align}}^{p}$, middle: the polarization geometry, and right: the polar plot of the molecular axis distribution.

Finally, we calculated the ${\text{LFPAD}}_{\text{align}}^{p}$ with a higher degree of alignments, $\left\langle {\text{cos}}^{2}\Theta \right\rangle =0.93$, where the values of *F_K_* for *K *=* *0, 2, 4, 6, 8, 10, 12, 14, 16, 18, 20, 22, and 24 are 0.0099, 0.0445, 0.0626, 0.0622, 0.0504, 0.0355, 0.0229, 0.0140, 0.0085, 0.0052, 0.0034, 0.0024, and 0.0022, respectively, as shown in Fig. 9. Even for such a higher degree of alignment, incoherent superposition of the ${\text{LFPAD}}_{\text{align}}^{p}$ from the nearly degenerate *I* 3*d* orbitals hinders the observation of the fine structures in the ${\text{LFPAD}}_{\text{align}}^{\text{sum}}$. Nevertheless, the fine structures become clearer in the ${\text{LFPAD}}_{\text{align}}^{p}$ from the 6*a*_1_, 3*b*_1_, and 3*b*_2_ orbitals. Thus, the observation of fine structures in the ${\text{LFPAD}}_{\text{align}}^{p}$ could be expected, if a molecular orbital without degeneracy is selected for a probe of the UXPD.

The present theoretical simulations indicate a favorable condition for the observation of fine structures in the ${\text{LFPAD}}_{\text{align}}^{p}$ in addition to the degree of alignment higher than 0.8: in the photoelectron energies, where the MFPADs can be interpreted as XPD profiles, the photoelectrons are mainly emitted in the polarization direction of the x-ray and then scattered at the atomic sites in the photoemission direction, so that the molecular structure is reflected in the MFPADs mainly through the interference terms between the directly emitted and singly scattered waves. Thus, sensitivity of the MFPADs to the molecular structure depends on the density distribution of the molecular orbitals from which the photoelectrons are emitted into the scattering sites. From this, a nondegenerate molecular orbital that meets this condition should be selected for the UXPD.

## V. OUTLOOK

As we discussed in Ref. 21, the interference Coulombic nature in the MFPAD makes it possible to track photodissociation or photoelimination over longer internuclear distances by UXPD, where time-resolved experiments that detect electronic structures are inapplicable. Such an advantageous aspect of UXPD is expected in tracking the elimination of the iodine atom from IPh molecule excited to the 3B_1_-diabatic state with a 266 nm laser pulse.^50^

**FIG. 10.**
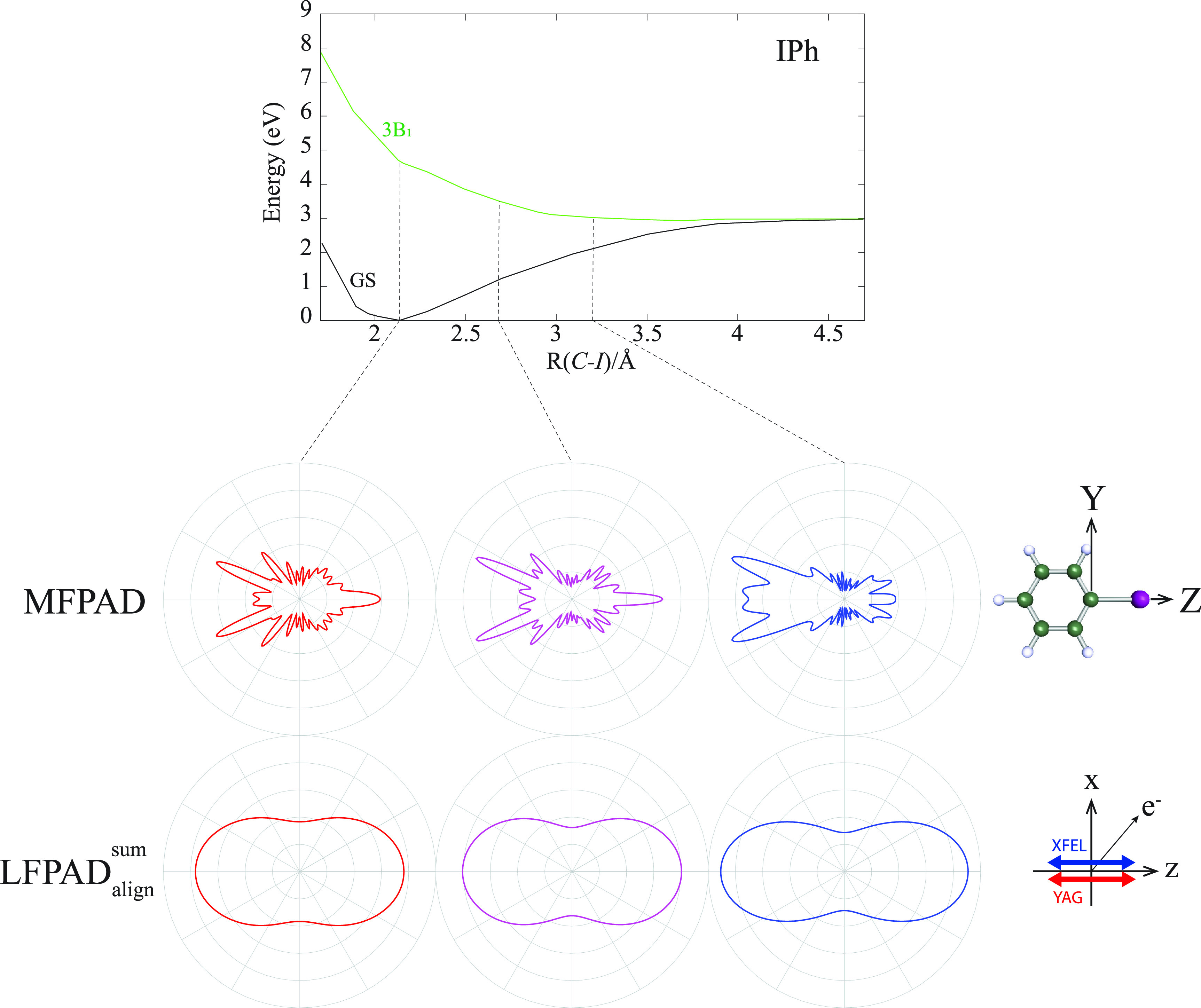
MFPADs and ${\text{LFPAD}}_{\text{align}}^{\text{sum}}$ of IPh depending on the *C*–*I* bond lengths. Potential energy curves of the ground and 3B_1_ states were taken from Ref. 50. The degree of alignment of $\left\langle \;{\text{cos}}^{2}\Theta \right\rangle =0.78$ was used in the calculations.

To know whether the ${\text{LFPAD}}_{\text{align}}^{\text{sum}}$ maintains the advantage or not, we calculated both the MFPAD and ${\text{LFPAD}}_{\text{align}}^{\text{sum}}$ for the *C*–*I* bond lengths of 2.7 and 3.2 Å, which are shown together with those calculated at the equilibrium bond length 2.1 Å, as shown in Fig. 10. The degree of alignment has been set to the same value as the present experiment $\left\langle {\text{cos}}^{2}\Theta \right\rangle =0.78$. To examine only the effects concerning the molecular geometry, we calculated both the MFPADs and ${\text{LFPAD}}_{\text{align}}^{\text{sum}}$ for the ground-state potential. The computed results demonstrate that both the MFPADs and ${\text{LFPAD}}_{\text{align}}^{\text{sum}}$ are insensitive to change in the *C*–*I* bond length near the equilibrium geometry (from 2.1 to 2.7 Å). This insensitivity is due to the contributions from photoelectrons emitted from the 1*a*_2_ and 7*a*_1_ orbitals through the pseudo degeneracy of the molecular orbitals to be ionized. However, when the *C*–*I* bond has been elongated by more than 1 Å than the equilibrium, the change of forward-focusing and backward-scattering effects is observed in the MFPAD, and they appeared in the ${\text{LFPAD}}_{\text{align}}^{\text{sum}}$ as the change of the XPD profiles as well, as shown in Fig. 10. Based on these theoretical predictions, we can summarize that the advantage of the MFPADs, which can track the temporal molecular geometries during photodissociation or photoelimination, is kept slightly in the ${\text{LFPAD}}_{\text{align}}^{\text{sum}}$ on the sum over the contributions from the degenerated orbitals under the currently achieved degree of alignment. Namely, for the larger change of molecular geometry of more than 1 Å, one can detect it through the change of the ${\text{LFPAD}}_{\text{align}}^{\text{sum}}$ profiles by UXPD.

## VI. CONCLUSION

In our previous work,^25,26,47^ we reported that a higher degree of alignment of sample molecules, $\left\langle \;{\text{cos}}^{2}\Theta \right\rangle >0.8$, is demanded to determine the molecular structures from XPD profiles for the aligned sample molecular ensembles. According to this criterion, we measured the *I* 3*d* XPD profile of IPh with $\left\langle \;{\text{cos}}^{2}\Theta \right\rangle =0.78$. However, we could not determine the local structure, i.e., the *C*–*I* bond length in the static ground-state IPh. This unexpected result is due to photoemission from the nearly degenerate molecular orbitals with respect to energy eigenvalues in the current experiment. The XPD profiles from the nearly degenerate five molecular orbitals contribute to the experimental ${\text{LFPAD}}_{\text{align}}^{3d}$, as discussed in Sec. IV B. Thus, intrinsic features of interference effects, which are observed in each XPD profile, are smeared out in the ${\text{LFPAD}}_{\text{align}}^{3d}$. If one selects a photoemission process from a nondegenerate molecular orbital, one can observe the intrinsic features of interference effects in the XPD profile, as demonstrated in the intensive calculations in Sec. IV B.

Finally, we summarize the criteria to perform the UXPD successfully as follows: (1) to use SXFEL, (2) to prepare sample molecules with the degree of alignments higher than 0.8, and (3) to select a photoemission process from a nondegenerate inner-shell orbital of the sample molecules, e.g., 1*s* orbitals of the second-row elements in the periodic table.

## Data Availability

The data that support the findings of this study are available from the corresponding author upon reasonable request.
